# Assessment of Anxiety in Pregnancy Following Assisted Reproductive Technology (ART) and Associated Infertility Factors in Women Commencing Treatment

**DOI:** 10.5812/ircmj.14465

**Published:** 2013-12-05

**Authors:** Chehreha Hashemieh, Leila Neisani Samani, Hamid Taghinejad

**Affiliations:** 1 Department of Midwifery, Faculty of Nursing and Midwifery, Ilam University of Medical Sciences, Ilam, IR Iran; 2 Department of Midwifery, Faculty of Nursing and Midwifery, Tehran University of Medical Sciences, Tehran, IR Iran; 3 Department of Nursing, Faculty of Nursing and Midwifery, Ilam University of Medical Sciences, Ilam, IR Iran

**Keywords:** Anxiety, Infertility, Reproductive Techniques

## Abstract

**Background:**

Successful pregnancy is the ultimate goal of almost all couples. However, this pleasant event is usually accompanied psychological and behavioral changes and can result in stress in women, particularly women who pregnant by assisted reproductive technology methods (ARTs).

**Objectives:**

This study aims to determine the anxiety level during pregnancy and its relation with infertility factors in women who has been pregnant by Assisted Reproduction Technology (ART) methods.

**Patients and Methods:**

A total number of 100 ARTs pregnant women who came to three infertility centers in Tehran from August to November 2009 participated in this descriptive cross sectional study. The rational for selecting the subjects was their availability to the researcher at the time of the research. Anxiety was measured by Beck Anxiety Inventory and for obtaining the infertility data, a questionnaire designed by the researcher was given to the subjects. Data were statistically analyzed using the inferential statistic of chi-square.

**Results:**

Study results showed that 34 % of subjects were anxious (moderate and sever levels in total). There are significant relations between infertility duration, history of treatment failure and anxiety level (P = 0.03) (P = 0.02). There were no statistically significant relationships with regard to other variables.

**Conclusions:**

Infertility duration and history of treatment failure in ARTs pregnant women are two factors that affect the anxiety level during pregnancy.

## 1. Background

Generally, successful pregnancy is the ultimate goal of almost all couples. However, this pleasant event is usually accompanied psychological and behavioral changes and can result in stress in women, particularly women who undergo ART (1). Assisted reproductive technology (ARTs) is one of the risk factors that can lead to anxiety in those who use these kinds of techniques (2). Recent research has shown that couples who try to have a child via ART techniques are perceived to have high levels of anxiety (3-6).

Anxiety, depression and fear of childbirth in 20 to 40% of infertile women are present as a result of Infertility investigations and the process of assisted reproduction technologies (ARTs). These symptoms of depression and anxiety will be resolved after successful treatments; however, the emotional burden of infertility will remain in almost 20% of women after birth (7).

Studies (8-12) showed that anxiety and depression during pregnancy can increase several complications such as spontaneous abortion, preeclampsia, preterm labor and low birth weight babies. In addition the babies of anxious women have more delayed development and low neurobehavioral scores. Further research showed that experiencing depressed mood or anxiety during pregnancy were also significant predictors of postpartum depression (13-17). Thus, evaluation of anxiety during pregnancy in general and in ART pregnancies in particular is important because of its side effects both for mothers and their child. Moreover, it is even necessary to eliminate stress for successful infertility treatments (18). Different studies have reported different factors causing mental stress particularly anxiety and depression in infertile cases using ART including uncertainty of the cause of infertility, uncertain treatment duration, financial stress, pressure from others who know the couples, and so forth (1, 3, 9, 19-21). In other words few consistent findings have been derived.

As stated earlier, ART, as one of the most prevalent ways of infertility treatments, can lead to anxiety. However because of the high rate of infertility an increasing number of infertile couples undergo ART (22). It can be said that the use of this method is much more prevalent in Iranian contexts due to the importance of having a child among Iranian families (6). That is, almost all of infertile couples in Iran ultimately try ART to cope with this crisis of their lives to prevent divorce (23).

Given the importance of controlling anxiety levels in pregnant women specially those experiencing infertility treatments and the prevalent use of ART in Iranian contexts, Psychological evaluation during pregnancy and recognizing major factors producing anxiety among Iranian infertile women using ART techniques is of great importance. Besides, in Iran few studies have been conducted to assess factors affecting the mental health of infertile patients; thus, little is known about mental health of Iranian infertile women. In fact a literature search found a limited number of articles in English addressing this subject in general and to date no other study have tackled the object of the present research.

## 2. Objectives

Considering what stated above as to the scarcity of research in this regard, inconsistency of findings and more importantly the significance of controlling anxiety levels, the aim of this study was to determine the anxiety level in Iranian ARTs pregnant women and factors influencing it including cause of infertility, numbers of treatment failure, different types of ART treatments, infertility duration, and history of treatment failure.

## 3. Material and Methods

### 3.1. Study Design

This study was conducted using a descriptive survey research design. Typically a questionnaire was used to determine the anxiety levels and its relation with infertility factors in women who get pregnant using Assisted Reproduction Technologies (ARTs). Data were collected from the sample population in a cross-sectional manner. Prior to the study the researcher informed the participants about the research objectives and took their written informed consent. The Ethics Committee of the Midwifery and Nursing department of Tehran University of medical sciences approves this study (at April 18, 2010 with approval NO: 791).

### 3.2. Subjects

A total number of 100 ARTs pregnant women who came to three infertility centers (Navid, Shayamehr, and Tehrani Nehad and Ashraf) in Tehran( capital of Iran) from August to November 2009 participated in this descriptive cross sectional study. It should be noted that we calculated the sample size based on statistical consulting: α = 0.05, β = 0.10, power = 0.90.

**Figure 1.**
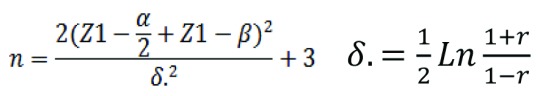
Equation

Inclusion criteria include: 1- Iranian women with gestational age from 8 to 42 weeks. 2- Not having any physical and psychological known illnesses during three past months. 3- Not having any sever crisis or stressful experiences such as death of close relatives or losing job, considering these items we excluded 5 people from the study process. The rational for selecting the subjects was their availability to the researcher at the time of the research. Sampling process was performed by convenience method.

### 3.3. Questionnaires

Anxiety measured by Beck Anxiety Inventory-21 (BAI) that developed by Beck et al. (24) to determine the frequency of anxiety symptoms in adults and adolescents. This tool can be answered by individuals and is easy to use (24). Individuals were requested to choose one of the following statements: “never”, “mildly”, “moderately”, and “severely” for every item. A score between 0 and 3 is given to the answers and the score range is 0-63. Individual who receive a high total score from the tool are experiencing a sever level of anxiety. According to the obtained scores, samples were divided in to the following groups: without anxiety (<9), mild (10 to 18), moderate (19 to 29), and sever (30 to 63). According to the authenticated textbooks, mild anxiety is considered to normal anxiety, so in this study we considered moderate and sever levels as anxious.

In term of Validity and reliability of Beck Anxiety Inventory-21 (BAI), it is noted that this instrument was used in several studies and its Validity and reliability was approved (25, 26). The anxiety questionnaire was answered by subjects in a quiet place and before the routine visit. For obtaining the infertility data, a questionnaire designed by the researcher.

### 3.4. Statistical Analysis

In order to analyze the findings of this study and determine the relationship between anxiety level and the specified variables of the study the inferential statistic of chi-square was used. To this end, SPSS computer package (SPSS version 14) was applied. The P value of <0.05 was considered statistically significant.

## 4. Results

In this study 100 ARTs pregnant women participated totally, results showed that 34% of subjects were anxious (Table 1). According to the findings of the study, infertility in 40% of subjects was due to male infertility, 30% to female, 14% to male & female and 16% was unexplained. Among participating Arts pregnant women, 43% used IVF, 39% ZIFT, 1% GIFT, 11% Egg donation, 2% Embryo transfer and 4% ICSI. Data suggested that there is significant relationships between infertility duration and anxiety level (P = 0.03). Findings in Table 2 support this point; that is, there was highest level of anxiety during 10 to 12 years of infertility and the mean score for infertility duration was 7.3 ±5.3. Also results indicated that there is significant relationship between history of treatment failure and anxiety level (P = 0.02). Additionally, findings showed that there was no statistically significant relationship with reference to other variables of the study including cause of infertility, numbers of treatment failure with the mean of 1.9 ± 1.6, and different types of ART treatments. Table 2 presents the quantitative data as to these factors. 

**Table 1.** Frequency of Anxiety Level in ART Pregnant Women

Anxiety Level	Frequency	%
**Without anxiety**	34	34
**Mild **	32	32
**Moderate **	23	23
**Sever **	11	11

**Table 2.** Relationship Between anxiety and Infertility Factors in ART Pregnant Women

	Without	Mild	Moderate	Sever	P value
Infertility duration (y)	Frequency (%)	Frequency (%)	Frequency (%)	Frequency (%)	0.03 ** X^2^ = 8.3
**1-3**	14 (43.8)	8 (25)	8 (25)	2 (6.2)	
**4-6**	6 (33.3)	10 (55.6)	2 (11.1)	0	
**7-9**	6 (28.6)	8 (38.1)	5 (23.8)	2 (9.5)	
**10-12**	0	3 (21.4)	6 (42.9)	5 (35.7)	
**13-15**	2 (50)	1 (25)	1 (25)	0	
**16-18**	2 (40)	0	1 (20)	2 (40)	
**≥19, One case without response **	3 (60)	2 (40)	0	0	
**Cause of ** **infertility**					0.8 X^2^ = 0.58
Male	16 (40.8)	11 (27.5)	9 (22.5)	4 (10)	
Female	10 (33.3)	10 (33.3)	6 (20)	4 (13.3)	
Male & female	4 (28.6)	4 (28.6)	4 (28.6)	2 (14.3)	
Unexplained	4 (25)	7 (43.8)	4 (25)	1 (6.2)	
**History of treatment failed**					0.02 ** X^2^ = 5.4
yes	15 (25)	21 (35)	17 (28.3)	7 (11.7)	
No	19 (47.5)	11 (27.5)	6 (15)	4 (10)	
**Number of treatment failed**					0.1 X^2^ = 1.8
1	1 (50)	1 (50)	0	0	
2	5 (17.2)	9 (31)	11 (37.9)	4 (13.8)	
3	5 (38.5)	7 (53.8)	0	1 (7.7)	
≥4, 40 cases without history of treatment failure	4 (25)	4 (25)	6 (37.5)	2 (12.5)	
**Kind of ART**					0.07 X^2^ = 5.2
IVF	18 (41.9)	11 (25.6)	9 (20.9)	5 (11.6)	--
ZIFT	8 (20.5)	14 (35.9)	11 (28.2)	6 (15.4)	--
GIFT	0	1 (100)	0	0	--
Egg donation	6 (54.5)	3 (27.3)	2 (18.2)	0	--
Embryo transfer	1 (50)	0	1 (50)	0	--
ICSI	1 (25)	3 (75)	0	0	--

## 5. Discussion

The result of this study showed that 0f 100 ARTs pregnant women that participated, 34% were anxious. Approximately, this percent constitutes one third of the total population under investigation. There is difference between the anxiety percent obtained from this study and those from previous ones. For example, the study of Dally et al. (14) indicated that 24.1% of spontaneous pregnant women were anxious (14). This difference can be associated to such factors which were the focus of this study, Presence of infertility history, frequent diagnosis tests, faced with large number of medical and surgical treatments, history of treatment failure and recent pregnancy.

The finding showed that there is significant relation between infertility duration and anxiety. This result is consistent with Ramezanzadeh et al. (27). The study of Ramezanzadeh (27) indicated that as the number of infertility years increases, the level of anxiety increases too. In her study she found that during 1 to 3 years of infertility, lower levels of anxiety have been observed while during 4 to 6 years anxiety level increases. The findings of the present study also showed highest level of anxiety during 10 to 12 years of infertility. These findings are probably indicative of the fact that as the number of infertility years increases, couples will be disappointed about having a child thus leading to higher levels of anxiety. Another reason may be the ageing phenomenon making them more anxious and disappointed about having a child. Furthermore, it may be associated to contextual factors like the reaction of their surrounding people which can make them more disappointed and thus leads to higher levels of anxiety in them.

Additionally, there was significant relation between history of treatment failure and anxiety. This result is consistent with Poikkeus et al. (7), Holter et al. (28) and Verhaak et al. (29, 30) (7, 28-30). One of the factors producing anxiety is failure of achieving the worthwhile and desired end. The degree of this failure controls the level of anxiety. When the anxiety increases as a result of failure, stress is produced (31). For subjects of this study, having a child was a worthwhile end. As a result of failure of achieving this end leads to anxiety in them. Other factors producing anxiety in these women are exposure to high level of hormones, side effects of medication (ovarian hyper stimulation syndrome), surgical operations and heavy costs of treatments. Although there is no relation between cause of infertility and anxiety, but the percent of anxious subjects in the both man and woman group was higher than the other groups. This can be associated to reason that the couples, both man and woman, are equally worry about the possibility of not having a child or failure of treatment.

There is no relation between number of treatment failed and anxiety but with the rise in failure numbers, there was a decrease in anxiety percent which can be associated to couples’ coping with this failure (29-31). If they cope with this failure effectively, they accept it as a reality in their life. Consequently they won't be disappointed and try to cope with the situation by changing their goals or treatment method.

According to the findings of this study indicating that 34% of subjects were anxious, it is suggested to use the Beck Anxiety Inventory or other valid questionnaires for anxiety screening in all ARTs pregnant women for identifying anxiety thus decreasing it or removing its side effects. Finding of the study also showed that there are significant relationships between history of treatment failure, infertility duration and anxiety level, thus there should be emotional support for these group of pregnant women in early detection of infertility, before starting the treatment cycle, and after pregnancy. It is also suggested that methods of anxiety control and management should be taught to physicians, midwives and personals of infertility centers.

Having not control group was the limitation of this study, hence Further research suggested to be conducted having a control group (spontaneous pregnant women), in order to comparing the results. Also according to the fact that infertility is a problem related to couples and affects different aspects of their life there is place for more research using 2 groups, one group spontaneous pregnant women with their husbands and other one ART pregnant women with their husbands, in order to compare them with each other. Findings of this study investigated anxiety levels related to fertility factors, there are needs to further researches that investigate the other ones. Literature review in Iran showed that majority of researches investigated anxiety in infertile women not in ART pregnant ones, but in our study we investigated this group in spite of lack of samples because of low rate of ART successfulness.

This study confirmed that depression and anxiety are common during ART pregnancies. In this study anxiety level in ART pregnant women was investigated with reference to variables like cause of infertility, numbers of treatment failure, and different types of ART treatments, infertility duration, and history of treatment failure. Based on the findings it was concluded that among these factors infertility duration and history of treatment failure in ARTs pregnant women are two factors that affect the anxiety level during pregnancy. In other words, there were higher levels of anxiety when the values for the history of treatment failure and infertility duration increase. Thus special attention to this group of pregnant women is necessary in order to control the effects of these factors on their mental health to decrease their anxiety levels.
